# Development of a Culture of Scholarship: The Impact of a Structured Roadmap for Scholarly Activity in Family Medicine Residency Program

**DOI:** 10.7759/cureus.7153

**Published:** 2020-03-01

**Authors:** Abdul Waheed, Munima Nasir, Erum Azhar

**Affiliations:** 1 Family Medicine, Wellspan Good Samaritan Hospital, Lebanon, USA; 2 Family and Community Medcine, Penn State University College of Medicine, Milton S. Hershey Medical Center, Hershey, USA; 3 Obstetrics and Gynecology, Maimonides Medical Center, Brooklyn, USA

**Keywords:** scholarship, scholarly activity, family medicine residency, community hospital, tertiary care, research

## Abstract

Introduction: The Accreditation Council of Graduate Medical Education (ACGME) mandates resident scholarship in all residency programs. Resident scholarship requirement continues to be one of the most common citations by the Residency Review Committee (RRC). This study evaluates the impact of a structured roadmap for resident scholarly activity in a single-family medicine residency program.

Methods: This retrospective study compares resident scholarship before and after exposure to a structured roadmap for scholarly activity as well as characteristics associated with higher scholarship productivity. The data was analyzed using Statistical Package for Social Sciences (SPSS Inc., Chicago, IL) version 16.0. Student’s t-test was used to calculate the statistically significant difference.

Results: There were a total of 16 residents who graduated in the first cohort whereas the second cohort consisted of 18 residents. There was a steady increase in resident scholarly activity over time. The number of publications by those who were exposed to a 13-step structured roadmap for scholarly activity was more than twice when compared with the first cohort. Those who pursued a fellowship after residency published three times more than those who did not.

Conclusion: Exposure to a structured roadmap for scholarly activity may be associated with higher production of resident scholarly activity. Larger studies comparing national level data from isolated community hospitals and big academic centers are needed for a conclusive argument. Although the availability of resources may increase the likelihood of more scholarship, the establishment of a research culture is more important. Further studies are needed to determine the factors which lead to the establishment of research culture in a residency program.

## Introduction

The Accreditation Council of Graduate Medical Education (ACGME) mandates resident scholarship in all residency programs [1]. Since 2006, it has been a hot topic of discussion with different authors suggesting various strategies to achieve a high level of resident scholarly activity in the residency program, including a point scoring system for scholarly activity to be introduced in the curricula [2-5]. It is an on-going challenge for the medical educators to get residents involved in scholarly activities [2,6]. Nationally, many family medicine residency programs struggle to fulfil these requirements every year. According to one report, this constituted as one of the most common citations by Residency Review Committee (RRC) in the year 2012 [3]. Crawford and colleagues reported that in 2009 only 12.5% of the programs had greater than 25% of their residents publish, and only 25.9% of the programs had 25% or more of their residents present at a regional, national, or international forum/medical conference [7].

National survey from 2011 looked at different factors [7]. Anecdotally, it is thought that residencies in a community hospital may have difficulty fulfilling these requirements as compared to tertiary care academic centers [5,8]. In community hospitals participation in practice based research network (PBRN) has been studied as one option by Weidner A et al. for residents [8]. In places where residents were involved in research projects with PBRN, the residents generate research questions, study design, analyze data and help with manuscript writing, though the study found more residency faculty involvement than residents in PBRN and proposes it as an excellent opportunity for residents education and scholarly activity [8].

A family medicine residency program sponsored by a large academic medical center was cited for not having enough scholarly activity in the year 2009. In 2009, a 13-step structured roadmap to finishing at least one scholarly activity by every resident was implemented as the intervention. With this shift, residents also had access to the academic tools and facilities that made formal research pursuits more obtainable. This presented a unique opportunity to study the production of scholarly work before and after through this retrospective analytical study.

## Materials and methods

This is a retrospective study that analyzed the results of two cohorts of residents in a single-family medicine residency program that implemented a structured roadmap for scholarly activity. The residency program retained a similar structure with more than 70% of the core faculty being the same as before. The first cohort was the graduates of the year 2007, 2008, and 2009. These residents completed their training before the implementation of the structured roadmap for scholarly activity. The second cohort, the graduates of the year 2010, 2011, and 2012, finished their residency while the structured roadmap was being implemented. A brief handbook was developed and made available to all residents for ready access to guidelines for scholarly activity. The 13-step structured roadmap is summarized in Table 1. 

**Table 1:** Summary of the 13-step structured roadmap to scholarly activity IRB: Institutional Review Board; CITI: Collaborative Institutional Training Initiative.

Step	Accomplishments	Deadline
Goals of Post Graduate Year-1: Prepare a project for IRB submission		
Step-0	CITI Training for Research Ethics during orientation and onboarding	End of July
Step-1	Review the “Resident Project Guidelines”	End of September
Step-2	Identify Your Specific Area of Interest	End of October
Step-3	Consider your Project Options and Potential Mentors	End of November
Step-4	Make Your Final Project Selection-Choose a Title	Early March
Step-5	Select a Project Mentor(s)	End of March
Step-6	Write the Project Proposal	End of April
Step-7a	Submit your Proposal for Mentor Approval	End of May
Step-7b	Submit your Proposal for scientific review	End of June
Goals of Post Graduate Year-2: Obtain IRB Approval, Collect, Enter, & Analyze Data		
Step-8	Submit your Proposal to IRB	End of July
Step-9	Begin Data Collection & Entry	End of September
Step-10	Complete Date Collection, Entry & Analysis	October-June
Goals of Post Graduate Year-3: Obtain IRB Approval, Collect, Enter, & Analyze Data		
Step-11	Write Abstract, Prepare for Presentation & Publication	End of July
Step-12	Schedule appointment with your advisor and Mentor for review	End of September
Step-13	Present at local, regional, national or international conference and submit for publication to a journal	October-June

The primary outcome measure was the number of peer-reviewed publications co-authored by the residents. The secondary outcome measures were trends in different types of publications by residents, and characteristics of residents (including the type of practice, additional training, medical school) publishing more than others overtime among both cohorts. 

Pubmed, Google Scholar, Worldwide Web, Doximity webpages were searched online to collect data on the outcome measures. This study was approved by the institutional IRB Committee from Human Subjects Protection Office as “Not Human Subjects Research”.

No sample size was calculated as all residents were included in the studied cohorts. Student’s t-test was used to compare the outcome measures using Statistical Package for Social Sciences (SPSS Inc., Chicago, IL) version 16.0.

## Results

**Figure 1:**
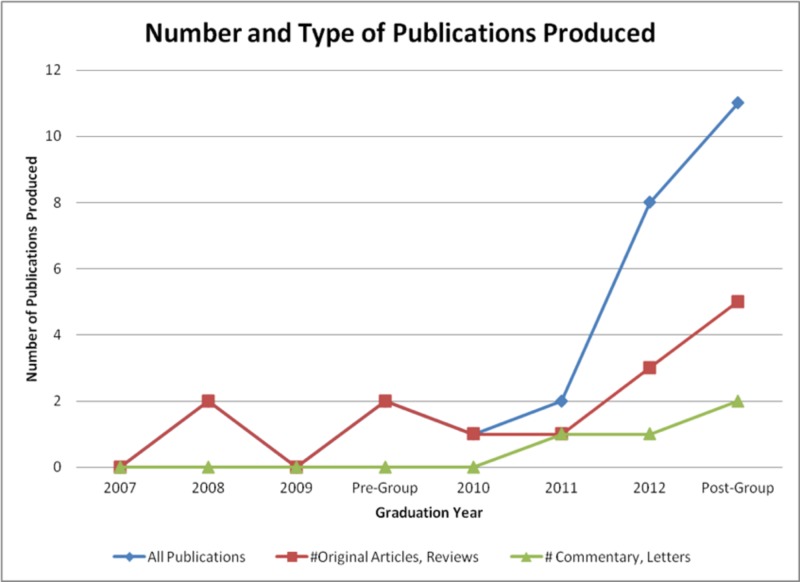
Overall trend of scholarly productivity as a function of time

There were a total of 16 residents who graduated in the first cohort whereas the second cohort consisted of 18 residents. Table 2 shows their demographic characteristics while Table 3 shows the number of total publications in each group with calculated p-value from student’s t-test for independent samples. Figure 1 shows overall increasing trend of resident authored publications over time. Table 4 shows calculated p-values for different characteristics overall among both cohorts. Those who pursued a fellowship after residency published three times more than those who did not. The number of residents were too small to calculate the t-score for each cohort individually and compare both.

**Table 2:** Baseline demographic characteristics of two cohort groups studied

Demographic		Cohort Group	
		Before the structured roadmap	After the structured roadmap
Gender	Male	8	9
	Female	8	10
Medical School Attended	LCME Accredited	2	5
	International	14	14
Additional Training/Fellowship	No	11	16
	Yes	5	3
Current setting of Practice	Non-academic	14	14
	Academic	2	5

**Table 3:** Impact of structured road map on scholarly productivity

p-0.21	Number of residents	Publications
Before the intervention	16	13 (0.8 per resident)
After the intervention	19	29 (1.5 per resident)

**Table 4:** Calculated p-values for different characteristics overall among both cohorts

Characteristics	p-value
LCME graduates publishing more than graduates from International medical schools	0.02
Those who pursued Fellowship published more than without any post residency training	0.01
Those who joined Academic practice post-residency published more than those in Non-academic Practice	0.5

## Discussion

Although the number of residents in two cohorts was not equal to each other, the distribution of characteristics overall is very comparable in both groups as shown in Table 2. The number of publications by those who were exposed to the structured roadmap were more than twice (total number 13 versus 29, publications per resident 0.8 versus 1.5) when compared with first cohort as shown in Table 2. The p-value (0.2) is not statistically significant because of the small number of residents in each group. As depicted in Figure 1, the resident scholarship showed continuously increasing trend with passing time. The maximum difference was seen in the year 2011 and 2012. This is because of either the long time required to publish from the beginning of a project or establishment of increasing culture of scholarship among residents as a small community. However, despite achieving an average of 1.5 publications per resident in the year 2012, the number of original articles per resident was still less than one. This possibly indicates that a culture of scholarship was getting stronger with time and the residency program might have just hit the critical mass required to take off in the year 2011 after availability and exposure to resources. It is well known that any social phenomenon in a closed community needs to reach a critical mass to become highly pervasive [9]. 

Fellowship post residency increased the likelihood of publishing by three times (p-value of 0.05) while the type of medical school attended and type of practice after graduation did not reach statistical significance. This may mean that future goals affect scholarly productivity more than the past background of medical education.

There are several potential limitations to this study including: 

1) The use of only peer-reviewed publications as surrogate for resident scholarly activity is a more stringent criteria when many other forms of scholarship which fulfill Glassick criteria are accepted in the graduate medical education as valid forms of scholarly activities. 

2) There are many potential confounders to studying the impact of a structured roadmap to scholarly activity with historical cohorts including: a) citation from the RRC at the ACGME served as a drive for the program leadership to work hard to cultivate a culture of scholarship, b) faculty development in scholarly activity was also conducted by the program leadership which would have added to the development of culture of scholarship, c) the GME office from the sponsor tertiary care medical center became increasingly involved in the administration of the program around the same time period which brought more academic resources to the program.

Scholarship is central to the work done by clinicians at academic medical centers. Although there is no universally accepted definition of scholarship, Boyer’s definition is widely accepted [3,10,11]. He proposed that a scholarly work involves multiple themes of knowledge acquisition and dissemination with four key components of scholarly activity: discovery, integration, application, and teaching [12]. Peer-reviewed publications are accepted as evidence of scholarship almost universally as mentioned by both Glassick et al. and Fincher et al. [11,13]. The introduction of scholarly activity point system in residency programs by Seehusen et al. was associated with an increase in the overall resident scholarly production, although it appeared to be more beneficial in programs that started with a low-baseline of scholarly activity [2].

## Conclusions

In summary, this study shows that the implementation of a structured roadmap for scholarly activity might be associated with higher production of resident scholarly activity and the development of a culture of scholarship in a program. Since this data is from only a single center which witnessed a transition, larger studies comparing national-level data from isolated community hospitals and big academic centers are needed for a conclusive argument. Although the availability of resources may increase the likelihood of more scholarship, further studies are needed to determine what leads to the establishment of research culture in a residency program than mere availability of resources.
